# Metastatic Renal Cell Carcinoma Detected by Postprandial Abdominal Pain: A Case Report

**DOI:** 10.1002/cnr2.70490

**Published:** 2026-02-09

**Authors:** Xinguang Wang, Nian Zhang, Shaowen Zeng

**Affiliations:** ^1^ Department of Urology The First Affiliated Hospital of Shandong Second Medical University WeiFang Shandong Province China; ^2^ Department of Anesthesiology The First Affiliated Hospital of Shandong Second Medical University WeiFang Shandong Province China

**Keywords:** endoscopic submucosal dissection, gastric metastasis, prognosis, renal cell carcinoma

## Abstract

**Background:**

Renal cell carcinoma (RCC), a prevalent malignancy in China, manifests annually in excess of 70 000 instances. At initial diagnosis, approximately one‐tenth of these patients present with metastatic disease. The predominant sites for distant dissemination encompass pulmonary, osseous, hepatic, and adrenal regions, whereas gastric involvement remains exceedingly rare. We clinically characterized a case by postprandially abdominal discomfort, which was endoscopic‐biopsy confirmed as a gastric tumor, and further diagnosis revealed the primary malignancy was kidney‐originated.

**Case:**

A 68‐year‐old male was admitted to our hospital for the evaluation of persistent postprandial abdominal pain and diarrhea that had persisted for 1 month. Gastroscopy revealed a gastric body tumor, leading to the performance of an endoscopic submucosal dissection (ESD). The initial pathological examination identified the tumor as gastric malignancy of undetermined origin. Concurrently, a comprehensive abdominal CT scan detected an additional renal tumor. Subsequent radical nephrectomy of the right kidney was performed, and the pathology confirmed the renal cell carcinoma shared the same origin as the gastric lesion. Over a follow‐up period of 38 months postoperatively, there has been no evidence of tumor recurrence or progression.

**Conclusion:**

Renal cell carcinoma has the potential to metastasize to the stomach, albeit this occurrence is not readily detectable in clinical practice unless it manifests as gastric symptoms. In instances where solitary superficial gastric metastasis is identified, aggressive surgical intervention can yield satisfactory clinical outcomes.

## 1 Introduction

Renal cell carcinoma (RCC) is one of the most common malignancies of the urinary system, accounting for approximately 2%–3% of all adult cancers [1]. It is characterized by a high degree of heterogeneity in terms of histology, genetic mutations, and clinical behavior [2]. Despite advances in early detection and treatment, RCC remains a significant cause of cancer‐related mortality due to its propensity for metastasis. The disease often progresses silently, with many patients presenting with advanced stages at diagnosis, underscoring the need for improved diagnostic and therapeutic strategies.

Metastasis in RCC typically involves the lungs, bones, liver, and brain [3]. However, gastrointestinal (GI) tract involvement, particularly gastric metastasis, is exceedingly rare [4]. When it occurs, gastric metastasis can present with nonspecific symptoms such as abdominal pain, nausea, vomiting, or gastrointestinal bleeding [5], which often mimic benign GI conditions. This rarity and the overlap of symptoms with common gastrointestinal disorders make timely diagnosis challenging, often leading to delayed intervention and poorer outcomes.

Previous studies on gastric metastasis from renal cell carcinoma have predominantly focused on cases presenting post‐nephrectomy or those with pre‐operative manifestations of gastric bleeding [6, 7, 8, 9]. The patient in this case initially presented with abdominal pain and diarrhea, without symptoms of hematemesis, bloody stools, or projectile vomiting. This report presents a rare case of RCC with gastric metastasis, highlighting the diagnostic challenges and clinical implications.

## 2 Case Report

**FIGURE 1:**
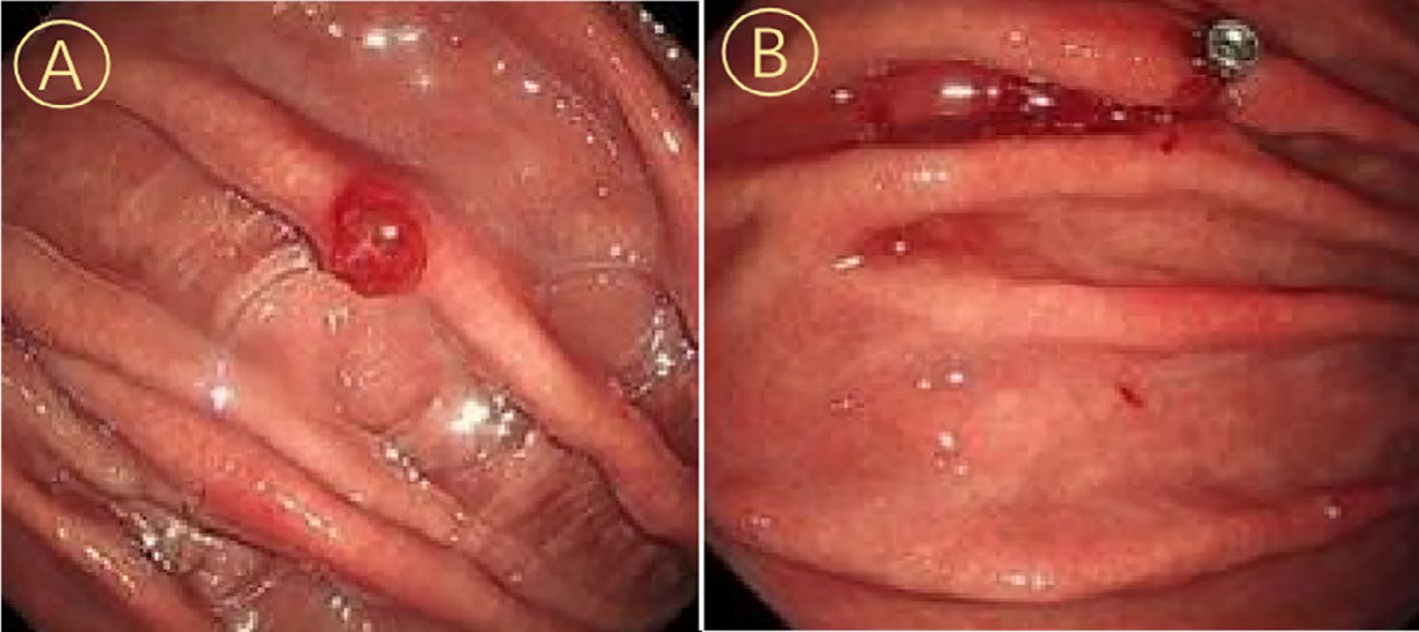
Endoscopic documentation of gastric body mass: Panel A (preoperative morphological characteristics) and Panel B (post‐ESD wound bed configuration).

**FIGURE 2:**
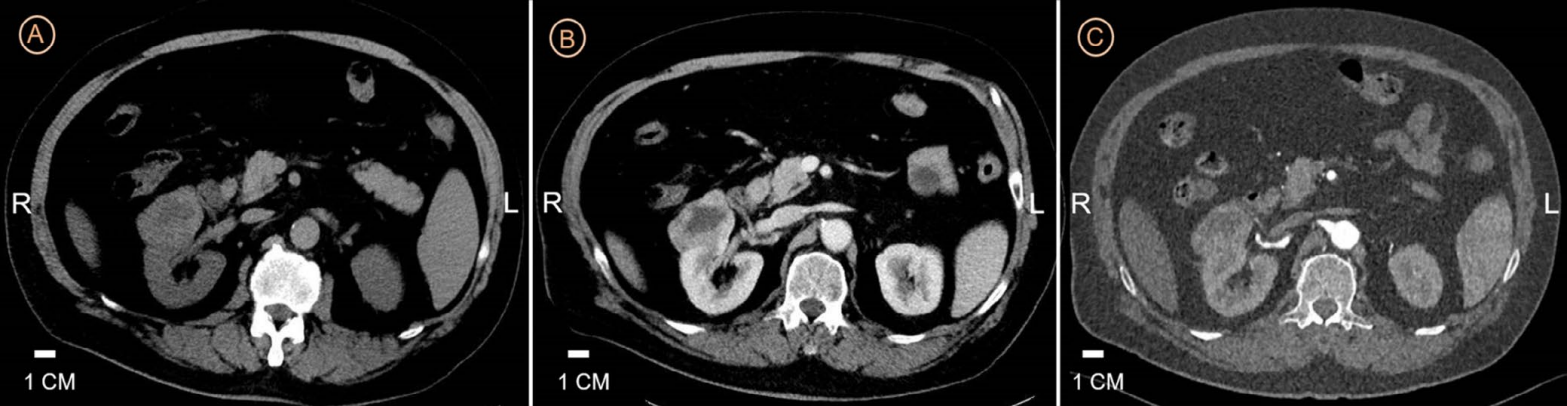
Axial CT images of the patient before radical nephrectomy, including (A) (plain axial CT), (B) (contrast‐enhanced axial CT in the arterial phase), and (C) (axial CT angiography, CTA).

A 68‐year‐old male presented to our Gastroenterology Department of The First Affiliated Hospital of Shandong Second Medical University with a one‐month history of postprandial abdominal pain and diarrhea in November 2021. His medical history included depression and coronary artery disease, with no prior diagnosis of diabetes or cerebrovascular events. Initial diagnostic gastroscopy identified erosive gastritis involving the gastric body and antrum, accompanied by a localized antral polyp. Endoscopic submucosal dissection (ESD) was performed for histological evaluation (Figure 1). Concurrent abdominal CT incidentally revealed a right renal mass, prompting multidisciplinary evaluation. Subsequent abdominal contrast‐enhanced CT and CT urography (CTU) confirmed a 5.1 × 4.2 cm heterogeneously enhancing right renal mass and a 2 cm right adrenal nodule (Figure 2).

**FIGURE 3:**
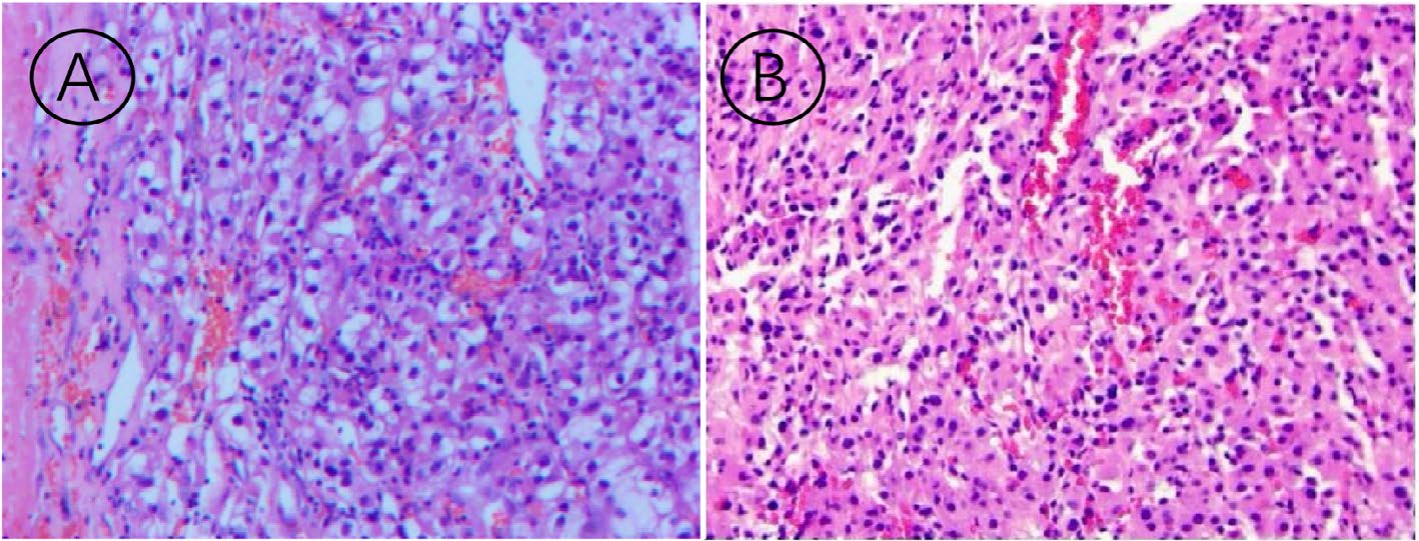
Hematoxylin and eosin (H&E) staining (×100). Panel A: Clear cell renal cell carcinoma of right kidney demonstrating geographic necrosis and hemorrhagic foci (WHO/ISUP grade 3), with capsular invasion (pT1bNxM1). Negative for neural/vascular invasion, renal pelvis involvement, and ureteral margin infiltration. Concurrent adrenal cortical nodular hyperplasia without malignant transformation. Panel B: Gastric metastatic focus exhibiting immunohistochemical positivity for pan‐cytokeratin (diffuse +) and vimentin (+).

Following comprehensive preoperative assessment excluding surgical contraindications, the patient underwent right radical nephrectomy with partial adrenalectomy. Histopathological examination demonstrated clear cell renal cell carcinoma (ccRCC) with capsular invasion without perinephric fat involvement, while the adrenal lesion showed benign cortical hyperplasia (Figure 3). Notably, histopathological correlation revealed morphological congruence between the gastric and renal lesions, demonstrating identical cytological features. This histoprofile constellation raised critical consideration of metachronous gastric metastasis from the primary renal cell carcinoma, despite the atypical presentation as a solitary lesion without concurrent systemic involvement. The subsequent management consisted of: (1) The first Follow‐Up (12 months Post‐Operation): The patient presented to the gastroenterology department with complaints of abdominal pain. Management included intestinal function regulation and modulation of the intestinal microbiota, following which the patient's condition improved, leading to discharge. (2) The second Follow‐Up (33 months Post‐Operation): The patient was admitted to the emergency department due to an episode of acute pancreatitis. Treatment involved pancreatic enzyme inhibition, gastroprotective therapy, and nutritional support. The patient's status improved, and was subsequently discharged. (3) The third Follow‐Up (Also at 33 months Post‐Operation): The patient experienced a recurrence of acute pancreatitis, which precipitated acute heart failure. Following emergency intervention, management included diuresis, anti‐inflammatory therapy, pancreatic enzyme suppression, and nutritional support. The patient's symptoms ameliorated, and the patient was discharged after stabilization. Postoperative surveillance over 38 months demonstrated no radiological or biochemical evidence of tumor recurrence.

**FIGURE 4:**
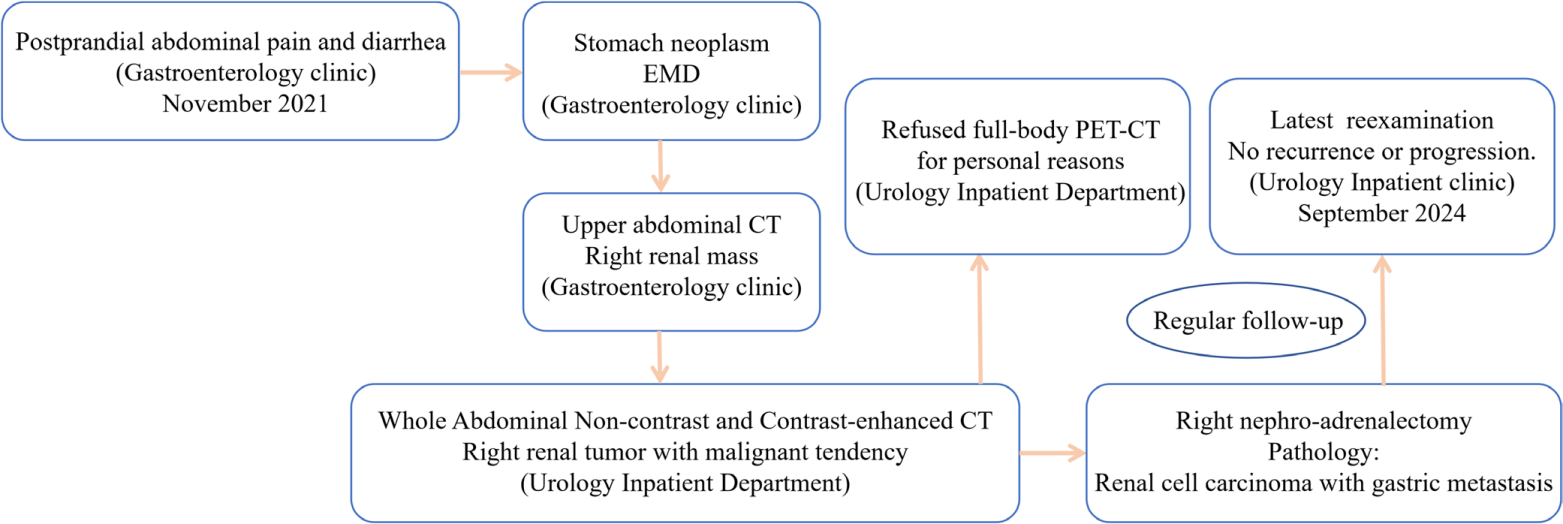
The diagnosis and treatment procedure of the case.

This case highlights three key clinical insights: (1) The diagnostic significance of incidental renal mass detection during abdominal imaging for non‐urological symptoms; (2) The favorable prognosis of ccRCC with negative surgical margins despite capsular invasion; (3) The importance of extended surveillance in elderly patients with complex medical histories. The extended disease‐free interval suggests complete oncological control despite initial multifocal presentations. The diagnosis and treatment process is shown in Figure 4.

## 3 Discussion

Renal cell carcinoma (RCC) exhibits a predilection for metastasis to sites such as the lungs, bones, liver, and lymph nodes [10], yet isolated gastric involvement is exceptionally rare (< 1% of cases). The majority of contemporary diagnoses are established incidentally through the widespread adoption of advanced abdominal imaging modalities, although affected individuals may present with either localized symptomatology or systemic clinical manifestations [1]. This case report presents a rare clinical entity of renal cell carcinoma with synchronous solitary gastric metastasis, successfully managed through radical nephrectomy and endoscopic submucosal dissection (ESD), achieving favorable outcomes with no recurrence during follow‐up.

The pathogenesis of gastric metastasis likely involves hematogenous dissemination, facilitated by the vascular richness of both organs. Clinically, nonspecific symptoms (e.g., abdominal pain or diarrhea) often mimic benign gastrointestinal disorders, delaying diagnosis [11]. In this case, endoscopic biopsy demonstrated a 6‐mm submucosal lesion with characteristic features, aligning with prior reports that > 70% of gastric RCC metastases are asymptomatic until advanced stages. The diagnostic accuracy of endoscopic ultrasound (EUS) for submucosal metastases is reported at 90%, yet preoperative misdiagnosis rates remain high at 40% due to overlapping imaging features with gastrointestinal stromal tumors (GISTs) or lymphomas [12]. Endoscopic evaluation and histopathology were pivotal in confirming metastatic RCC, emphasizing their indispensable role in patients presenting with gastrointestinal complaints.

Management of solitary gastric metastases lacks standardized protocols. Localized resection—surgical or endoscopic—is preferred for accessible lesions, aiming for complete tumor eradication. Endoscopic resection, as performed here, offers minimal invasiveness and rapid recovery, particularly suitable for superficial tumors [13]. Emerging evidence supports its efficacy in select cases, correlating with prolonged survival and symptom relief. A cohort study of 86 patients with metastatic renal cell carcinoma (mRCC) demonstrated that prolonged overall survival was significantly associated with lung‐limited metastatic burden, absence of locoregional tumor recurrence, and extended disease‐free intervals following primary tumor resection [14]. In contrast, systemic therapies (e.g., TKIs, immunotherapy [15, 16]) are typically reserved for metastatic RCC. In the latest proof‐of‐concept investigation, prostate‐specific membrane antigen (PSMA)‐guided metastasis‐directed therapy (MDT) demonstrated robust and durable oncologic efficacy in patients with oligometastatic RCC, even at a 5‐year follow‐up [17].

A major challenge in the clinical management of this patient was suboptimal compliance; persistent dietary irregularities exacerbated the gastrointestinal burden, likely contributing to recurrent episodes of acute pancreatitis and subsequent acute heart failure. Furthermore, the absence of regular adjuvant therapy following surgery heightened the risk of disease recurrence and metastatic progression of renal cell carcinoma. The KEYNOTE‐564 study indicated that Pembrolizumab reduces the risk of disease recurrence or death by 32% [18]. Furthermore, the FDA approved the combination of Axitinib and Pembrolizumab for the first‐line treatment of metastatic RCC as early as 2019. We have further consulted relevant literature (EAU Guidelines 2022 [19]), which also noted Pembrolizumab for the adjuvant treatment of metastatic RCC. It is important to note that in China, the combination of Toripalimab and Axitinib is currently recommended for the first‐line treatment of advanced RCC. Pembrolizumab, due to its high cost and lack of coverage (fully out‐of‐pocket in China), was declined by this patient. The patient demonstrated poor compliance, failing to adhere to the recommended follow‐up schedule (especially every 3 months for the first 2 years) and not undergoing systematic targeted or immunotherapy. The two episodes of acute pancreatitis at 33 months post‐operation further exacerbated the patient's treatment burden.

This case reinforces the need for multidisciplinary collaboration and lifelong surveillance in RCC patients. While endoscopic resection proves valuable for localized gastric metastases, further research is warranted to optimize diagnostic algorithms and therapeutic guidelines for such rare presentations. Clinicians must maintain a high suspicion index to ensure timely intervention and improve prognoses in this challenging clinical scenario.

## 4 Conclusion

This article provides a comprehensive analysis of the clinical manifestations associated with gastric metastasis from renal cell carcinoma and emphasizes the critical importance of endoscopic mucosal biopsy in diagnosing such cases. This case highlights the critical importance of a thorough diagnostic workup for any suspicious mass detected in patients presenting with gastrointestinal symptoms. Furthermore, it underscores the necessity of subsequent comprehensive systemic evaluation to guide appropriate clinical management.

## Author Contributions

All authors have made substantial intellectual contributions to this work. Xinguang Wang was responsible for the conception of the study, data acquisition and analysis, and drafting the initial manuscript. Nian Zhang participated in data interpretation and critically revised the manuscript for important intellectual content. Shaowen Zeng supervised the project, provided final approval of the version to be published, and is the corresponding author. All authors read and approved the final manuscript.

## Funding

The authors have nothing to report.

## Ethics Statement

Ethical approval for this case report was obtained from the Institutional Ethics Committee of The First Affiliated Hospital of Shandong Second Medical University. The study was performed in accordance with the ethical standards as laid down in the 1964 Declaration of Helsinki and its later amendments.

## Consent

Written informed consent was obtained from the patient for publication of this case report and any accompanying images. A copy of the written consent is available for review by the Editor‐in‐Chief of this journal.

## Conflicts of Interest

The authors declare no conflicts of interest.

## Data Availability

The data that support the findings of this study are available on request from the corresponding author. The data are not publicly available due to privacy or ethical restrictions.
